# Infection à VIH-2 au Sénégal: échecs virologiques et résistances aux antirétroviraux (ARV)

**DOI:** 10.11604/pamj.2019.33.222.15771

**Published:** 2019-07-18

**Authors:** Selly Ba, Ndeye Mery Dia-Badiane, Stephen Edward Hawes, Louise Fortes Deguenonvo, Fatima Sall, Cheikh Tidiane Ndour, Khadim Faye, Fatou Traoré, Macoumba Touré, Marie Pierre Sy, Dana Noelle Raugi, Nancy Berenice Kiviat, Robert Alexander Smith, Moussa Seydi, Papa Salif Sow, Geoffrey Scott Gottlieb

**Affiliations:** 1 Service des Maladies Infectieuses CHUN de Fann, Dakar, Sénégal; 2 University of Washington, Seattle, USA

**Keywords:** VIH-2, échecs virologiques, résistances génotypiques, HIV-2, Virologic failures, genotypic resistance

## Abstract

**Introduction:**

Le VIH-2, endémique en Afrique de l'Ouest, est naturellement résistant aux inhibiteurs non nucléosidiques de la rétro transcriptase (INNRTI), ce qui rend difficile la prise en charge dans les pays en développement. L’objectif ici était de déterminer la prévalence de l'échec virologique au 12^éme^ et 24^éme^ mois (M12 et M24) de traitement antirétroviral de première ligne chez les patients infectés par le VIH-2 et d'en décrire les résistances génotypiques associées.

**Méthodes:**

Il s'agit d'une étude descriptive longitudinale et prospective, durant la période de novembre 2005 à juin 2017. L'échec virologique a été défini comme toute charge virale supérieure à 50 copies/ml après 6 mois de traitement ARV à deux reprises. La recherche de mutations de résistance a été réalisée dans les régions codantes de la protéase et de la transcriptase inverse.

**Résultats:**

Au total 110 patients ont été colligés, d'âge médian de 46 ans (Extrêmes 18-67) avec un ratio F/H de 2,54. À l'inclusion, la charge virale était détectable dans 44% des cas avec une médiane de 935cp/ml (Extrêmes 17-144038). Le schéma antirétroviral associait 2 INTI à 1IP dans 94% des cas. La durée médiane de suivi était estimée à 1200 jours (Extrêmes 1-3840). 94 puis 76 patients ont respectivement complété leur bilan à M12 et M24. Au suivi M24, 39 patients étaient en échec virologique soit une prévalence de 39% estimée à 33% à M12 et 11% à M24. 45% des patients avaient des résistances aux inhibiteurs nucléosidiques de la transcriptase inverse (INTI), 41% des résistances aux IP et 30% des multi résistances aux INTI et IP.

**Conclusion:**

Il est impératif de rendre accessible les nouvelles classes thérapeutiques pour le traitement de sauvetage des patients infectés par le VIH-2 dans les pays à ressources limitées.

## Abstract (en)

**Introduction:**

HIV-2, endemic in West Africa, has a natural resistance to non-nucleoside reverse transcriptase inhibitors (NNRTIs) which makes it difficult to treat it in developing countries.

**Methods:**

We conducted a descriptive, longitudinal, prospective study over the period November 2005-June 2017. Virologic failure has been defined as any viral load greater than 50 copies/ml after 6 months of ARV treatment administered twice. Assays for detecting drug-resistance mutations was performed in the protease-coding region and in the reverse transcriptase-coding region.

**Results:**

Data from a total of 110 patients were collected. The patients had a median age of 46 years (ranging from 18 to 67) with a sex-ratio F/M of 2.54. At inclusion, viral load could be assessed in 44% of cases with a median of 935cp/ml (ranging from 17 to 144038). Antiretroviral regimen consisted of a combination of 2 NRTIs and 1IP in 94% of cases. The median follow-up was 1200 days (ranging from 1 to 3840); 94 then 76 patients completed their 12-month and 24-month assessments respectively. At 24-month follow-up, 39 patients had virologic failure, reflecting a prevalence of 39% estimated at 33% at 12-month follow-up and at 11% at 24-month follow-up; NRTIs resistance was observed in 45% of patients, IP resistance in 41% of patients while multi-NRTIs resistance and multi-IP resistance in 30% of patients.

**Conclusion:**

Currently, there is an urgent need to make available the new therapeutic classes of ARV for second line ART for patients living with HIV-2 with therapeutic failure in resource-limited settings.

## Introduction

Le VIH-2 est endémique en Afrique de l'ouest avec une diffusion limitée dans d'autres lieux qui ont des liens socio-économiques avec cette région [1]. Environ 1 à 2 millions de personnes sont infectées par le VIH-2 dans le monde [2]. En comparaison avec le VIH-1, le potentiel évolutif de l'infection VIH-2 est plus lent que celui de l'infection à VIH-1, avec une réplication virale moins importante, une latence clinique asymptomatique plus longue, une diminution du taux de lymphocytes TCD4 plus lente, une charge virale plasmatique plus faible, une survie plus longue et une transmission de la mère à l'enfant plus faible [3, 4]. Néanmoins, toutes les manifestations cliniques observées au cours de l'infection par VIH-1 ont été rapportées au cours de l'infection à VIH-2: primo-infection, infections opportunistes et néoplasies. De même, l'infection à VIH-2 peut conduire à un SIDA clinique et au décès. Afin d'améliorer leur survie, les patients vivant avec le VIH-2 peuvent bénéficier d'un traitement antirétroviral [5, 6]. Dans le domaine du traitement antirétroviral, les choix thérapeutiques sont plus limités pour le VIH-2 que pour le VIH-1, le VIH-2 est naturellement résistant aux inhibiteurs non nucléosidiques de la transcriptase inverse (INNTI), à l'enfuvirtide, inhibiteur de fusion (T-20) et à certains inhibiteurs de la protéase (IP) [5]. Ce qui a motivé les recommandations de l'OMS d'utiliser les IP associés aux inhibiteurs nucléosidiques de la transcriptase inverse (INTI) comme traitement de première intention [7]. Leur application pourrait avoir comme corolaires des difficultés de traitement de deuxième ligne dans les pays en voie de développement. Ce d'autant plus, la résistance croisée phénotypique dans la classe des IP ainsi que des INTI constitue une problématique pour le traitement de l'infection à VIH-2 [8, 9]. C'est dans ce contexte que s'est inscrite notre étude qui s'est fixée comme objectifs: de déterminer les échecs virologiques à M12 et M24 de traitement antirétroviral de première ligne; d'en décrire les résistances génotypiques associées.

## Méthodes

Notre travail a eu pour cadre d'étude le Service des Maladies Infectieuses Ibrahima Diop Mar (SMIT) du CHUN de Fann qui est la structure de référence nationale de prise en charge du VIH. Il s'agit d'une étude descriptive, prospective et longitudinale allant de la période de novembre 2005 à juin 2017. Les critères d'inclusion étaient le fait d'être infecté par le VIH-2, âgé de 18 ans et plus, consentant à participer à l'étude, naïf et éligible au traitement antirétroviral selon les critères de l'Initiative Sénégalaise d'Accès aux Antirétroviraux (ISAARV) ou déjà sous traitement antirétroviral. Les critères de L'ISAARV étaient le fait d'être symptomatique, quel que soit le taux de lymphocytes TCD4, pauci symptomatique avec un taux de CD4 inférieur ou égal à 350 cellules/mm^3^ ou asymptomatique avec un taux de CD4 inférieur à 200 cellules/mm^3^. Lesquels critères étaient revus en 2013 préconisant l'initiation du traitement ARV au seuil de lymphocytes CD4 inférieur à 500 cellules/mm^3^ [10]. Le diagnostic avait été fait par le Determine (Alere Determine TM HIV-1-2) qui est un test immunologique qualitatif in Vitro à lecture visuelle pour la détection des anticorps anti VIH-1 ou VIH- 2 dans le sérum, le plasma ou le sang total humain et par l'Immunocomb II HIV- 1-2 bispot (Orgenics) qui est un test rapide de dépistage et de différenciation des anticorps dirigés contre le VIH-1-2. Les critères de non inclusion étaient le fait d'être co-infecté par le VIH-1 et 2. Après l'inclusion un suivi clinique, immunologique et virologique était régulier, au premier mois et tous les quatre mois. L'échec virologique a été défini comme toute charge virale supérieure à 50 copies/ml à deux reprises après six mois de traitement antirétroviral [11].

La quantification de la charge virale a été faite par extraction de l'acide ribonucléique (ARN) virale plasmatique et amplification par polymerase chain reaction (PCR). Les produits amplifiés ont été détectés en utilisant l'instrument Abbott m2000. Les niveaux d'ARN du VIH-2 ont été calculés en utilisant le logiciel LDA m2000rt [11]. La recherche des mutations de résistances a été faite dans les régions codantes de la protéase et de la transcriptase inverse par amplification par PCR (QIAamp Viral RNA Minikit) [12] et séquençage selon la technique du séquençage génétique de Sanger comme décrit par Raugi D [12]. Des méthodes du maximum de vraisemblance ont été utilisées pour estimer les arbres phylogénétiques. Les séquences de VIH-2 ont été réparties en groupes sur la base de ces analyses phylogénétiques [12]. Les numéros d'accès GenBank des séquences de VIH-2 de cette étude étaient FJ812523 à FJ812621, FJ812624 à FJ812692 et KC768350 à KC768705 [12]. Le recueil des données a été fait à l'aide de questionnaires préétablis qui avaient permis de renseigner les paramètres suivants: données sociodémographiques (âge, sexe), données cliniques (Indice de masse corporelle (IMC) initial, stade OMS, porte d'entrée aux soins du VIH), données para cliniques (taux de CD4, charge virale ( CV), données thérapeutiques (les schémas thérapeutiques ARV), données évolutives (l'IMC annuel, le taux de lymphocytes TCD4 annuel, la CV annuelle, les perdus de vue et les personnes décédées). Toutes les données ont été saisies et analysées à l'aide des logiciels Excel et Epi Info 2002. Les statistiques descriptives de fréquence et de moyenne ont été utilisées. Les moyennes ont été comparées à l'aide des tests de Student et de Kruskal Wallis en respectant leur condition d'applicabilité. Une p value inférieure à 0,05 a été considérée comme statistiquement significatif. L'étude a été autorisée par les comités d'étude institutionnels de l'Université de Washington (IRB00000242) et par le Comité éthique national Sénégalais/Ministère de la santé (IRB00002659). Le consentement éclairé des patients était recueilli à travers un formulaire de consentement. Une base de données anonyme a été constituée à partir des questionnaires renseignant sur les caractéristiques sociodémographiques et les données médicales des patients. Aucune information ne permettait d'identifier les patients inclus dans cette étude. La base de données reste une propriété de l'Université de Washington et de la clinique des maladies infectieuses de Fann.

## Résultats

**Caractéristiques des patients à l'inclusion:** au total 110 patients ont été colligés, d'âge médian de 46 ans avec des extrêmes de 18 et 67 ans; la majorité était de sexe féminin (79) avec un ratio F/H de 2,54. À l'inclusion, 76% des patients étaient symptomatiques, classés au stade 4 de l'OMS (31%), au stade 3 (45%); 24 (22%) étaient au stade 2 et 2 (02%) au stade 1. L'indice de masse corporelle (IMC) médian au moment de l'examen initial était de 20,20 kg/m^2^ avec des extrêmes de 10,30 et 35,15 kg/m^2^. Des infections opportunistes étaient présentes chez 69 patients. Ces infections étaient dominées par les affections cutaneo-muqueuses dans 63% des cas à type de dermatose (15 patients), de candidose vaginale (20 patients), de zona (05 patients), d'herpès génital (3 patients). Les affections digestives suivaient (30% des cas), représentées par une candidose digestive (10 patients), une diarrhée chronique (10 patients). La tuberculose pulmonaire était présente chez 5 patients. L'immunodépression était globalement sévère avec un taux médian de lymphocytes T CD4+ de 225 cellules/mm^3^ (Extrêmes: 6- 1003). À peu près la moitié (48%) avait un taux de CD4 inférieur à 200 cellules/mm^3^ (53 patients), 31% avait un taux compris entre 200 et 350 cellules/mm^3^ (34 patients), 12 patients soit 11% un taux compris entre 350 et 500 et seul 10% avait un taux supérieur à 500 cellules/ mm^3^ (11 patients). Sur le plan virologique, 100 patients ont eu à bénéficier à l'inclusion d'une évaluation de la CV, parmi eux, 44 soit 44% patients avaient une charge virale détectable avec une CV médiane de 935 copies/ml de sang (Extrêmes: 17 -144038). Chez les patients naïfs qui commençaient le traitement ARV de première ligne et qui étaient au nombre de 68, la CV disponible pour 59 patients était détectable chez 31 patients (45%) avec une médiane de 1272 copies/ml de sang (Extrêmes: 17- 144038). Le schéma antirétroviral associait 2INTI à 1IP dans 94% des cas (104/110). Sur les 110 patients, 58 (52%) ont débuté un traitement à base d'Indinavir non boosté (IND, seul IP disponible aux débuts de l'ISAARV, avant d'être mis sous Lopinavir/Ritonavir (LPV/R), rendu disponible par l'ISAARV en 2008. Les combinaisons prescrites étaient à base de AZT/3TC/IND (51 patients), TDF/3TC /IND (1 patient), D4T/3TC/IND (5 patients), DDI/3TC/IND (1 patient), 46 patients avaient eu à bénéficier d'emblée d'un régime à base de LPV/R, avec comme associations AZT/3TC/LPV/R (40 patients), TDF/3TC/LPV/R (4 patients), D4T/3TC/LPV/R (2 patients). Un régime à base de 3 INTI était institué chez 6 patients (Tableau 1).

**Tableau 1:** caractéristiques des patients à l’inclusion

Variables	Effectifs	Pourcentage
**Sexe**		
Masculin	31	(28%)
Féminin	79	(72%)
**Age médian (Extrêmes)**	46 ans	(18-67)
**Indice de Masse Corporelle (IMC) IMC médian (Extrêmes)**	20,20 kg/m^2^	(10,30-35,15)
**Classification selon l’Organisation Mondiale de la Santé (OMS)**		
Stade 1	2	(02%)
Stade 2	24	(22%)
Stade 3	50	(45%)
Stade 4	34	(31%)
**Taux médian de T CD4 (Extrêmes)**	225 cellules/mm^3^	(6 -1003)
**Charge virale détectable(CV)** **CV médiane (Extrêmes)**	44% 935 copies/ml	(17-148033)
**Schéma thérapeutique initial**		
AZT-3TC-IND	51	46,40%
D4T-3TC-IND	5	4,50%
TDF-3TC-IND	1	0,9%
DDI-3TC-IND	1	0,9%
AZT-3TC-LPV/R	40	36,40%
TDF-3TC-LPV/R	4	3,60%
D4T-3TC-LPV/R	2	1,80%
TDF-3TC-ABC	5	4,50%
AZT-3TC-ABC	1	0,9%

**Aspects évolutifs:** la durée médiane de suivi était de 1200 jours avec des extrêmes de 1 à 3840 jours. Au total, 94 puis 76 patients ont respectivement complété leur bilan de M12 et M24 de suivi. Parmi eux, 68 patients n'étaient pas prétraités et recevaient le traitement de 1^ère^ ligne pour la première fois, 56 puis 50 d'entre eux ont complété leur suivi à 12 mois et à 24 mois. Au cours du suivi, sur le plan clinique, chez la totalité des patients un gain pondéral de 1,10 Kg/m^2^ a été noté la première année (n= 94), par contre la deuxième année il y avait eu une légère baisse de l'IMC médian de 0,32 Kg/m^2^ (n= 76). Chez les patients qui étaient déjà sous traitement ARV à l'inclusion (n= 42), une baisse de l'IMC médian de 0,03 Kg/m^2^ a été notée à M12 (n= 38) et un gain de 0,05 Kg/m^2^ à M24 (n=26). Alors que chez les patients non prétraités qui recevaient le traitement de 1^ère^ ligne pour la première fois un gain pondéral médian de 1,2kg/ m^2^ a été noté à M12 (de 19,08 Kg/m^2^ à l'inclusion l'IMC médian est passé à 20,32 kg/m^2^ à M12 (n= 56)), par contre à M24, un gain moindre de 0,24Kg/m^2^ de l'IMC médian a été noté (n = 50).

**Figure 1:**
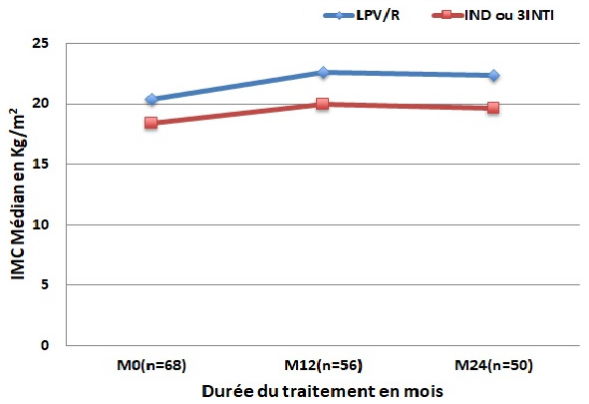
évolution du taux médian de l’Indice de Masse Corporelle (IMC) chez les patients qui commençaient le traitement de 1^ère^ ligne

**Figure 2:**
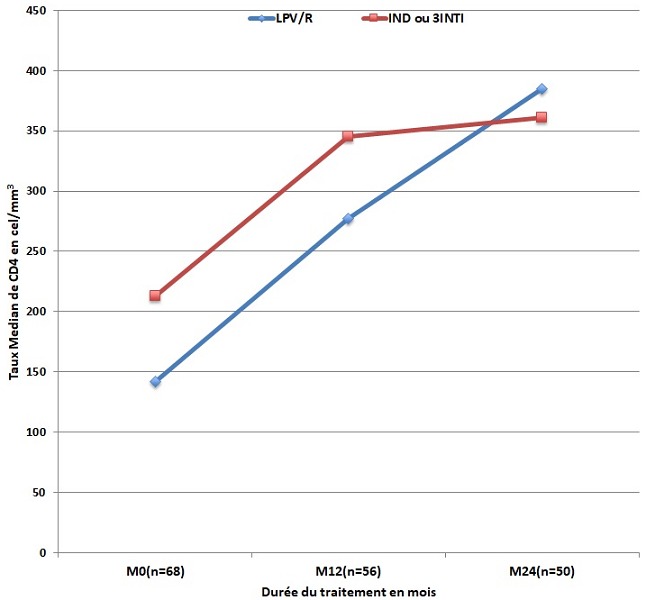
évolution du taux médian de lymphocytes CD4 chez les patients qui commençaient le traitement de 1^ère^ ligne

L'évolution de l'IMC corrélée avec le schéma antirétroviral chez ces patients non prétraités avait montré un gain pondéral légèrement plus accentué chez les patients qui avaient bénéficié d'un schéma antirétroviral à base de LPV/R (de 20,36 Kg/m^2^ à l'inclusion (n=36). Il est passé à 22,59Kg/m^2^ soit un gain de 2,23 Kg/m^2^ la première année (n= 27) alors que pour ceux qui étaient sous régime non boosté (IND ou 3 INTI) (n=32 à l'inclusion), le gain était de 1,18 Kg/m^2^ (18,42 à 20 Kg/m^2^) la première année (p= 0,18) (n=29). À la deuxième année, une baisse de l'IMC médian de 0,12Kg/m^2^ était notée chez ceux qui étaient sous LPV/R (n=24) et de 0,34 kg/m^2^chez les autres (n=26) (p= 0,04) (Figure 1). Sur le plan immunologique, chez la totalité des patients le taux médian de lymphocytes CD4 est passé de 217 cellules/mm^3^ à l'inclusion à 312 cellules/mm^3^ à M12 et à 380 cellules/mm^3^ à M24 soit un gain respectif de 95 et 68 cellule/mm^3^. Chez les patients qui étaient déjà soumis à un traitement ARV à l'inclusion, un gain immunologique de 73 cellules/ mm^3^ a été noté à M12 et un gain de 65 cellules/ mm^3^ à M24. Chez les patients non prétraités, ce gain était de 114 cellules/ mm^3^ à M12 et de 78 cellules/ mm^3^ à M24. Chez ces derniers, cette évolution immunologique corrélée avec le schéma antirétroviral a été pratiquement la même la première année comparativement au régime sous IP boosté (n = 27) ou sous (IND ou 3INTI) (n=29) soit un gain de 130 cellules/mm^3^, par contre au 24 mois de suivi le gain a été plus important chez les patients sous LPV/R (108 (n = 24) versus 16 (n = 26) p = 0,32 (Figure 2). Sur le plan virologique, d'une manière générale la charge virale (CV) était détectable à plus de 50 cp/ml chez 39 patients prétraités ou non soit une prévalence totale de 39% (39/100) estimée à 33% à M12 (31/94) et 11% (8/76) à M24. Chez les patients prétraités, la prévalence de l'échec virologique était de 36% à M12 (14/38) et de 3,8% à M24 (1/26). Chez les patients non prétraités, la proportion de ceux qui avaient un taux de CV détectable à plus de 50cp/ml était de 17/56 à M12 et de 7/50 à M24 soit une prévalence respective d'échec virologique de 30% et 12%.

**Figure 3:**
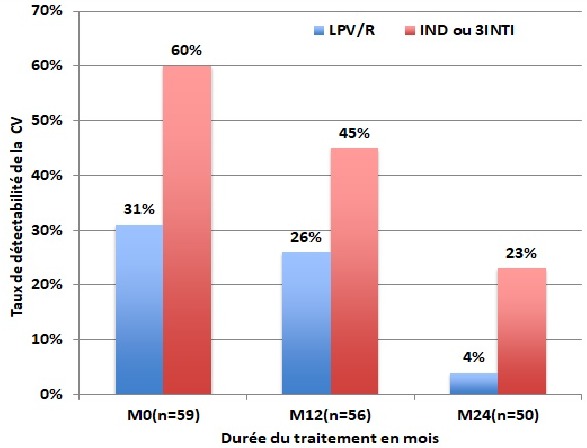
évolution du taux de détectabilité de la charge virale (CV) chez les patients qui commençaient le traitement de 1^ère^ ligne

La CV corrélée avec le schéma thérapeutique a montré chez ces patients non prétraités une meilleure réponse virologique chez les patients sous IP boosté. La proportion de patients qui avaient une charge virale détectable est passée de 31% (9/29) à l'inclusion à 26% (7/27) à M12 chez les patients sous IP boosté et de 60% (18/30) à (45%) (13/29) chez ceux qui étaient sous (IND ou 3INTI) à M12 (p=0,019. De même, la deuxième année de suivi on avait noté 4% (1/ 24) d'échecs chez les patients sous IP boosté versus 23% (6/26) chez les patients sous (IND ou 3INTI) (p=0,32) (Figure 3). Ces échecs virologiques dans leur globalité ont été associés des résistances génotypiques aux INTI et aux IP. Quarante-quatre (44 patients) soit 40% de la cible avaient eu à bénéficier des tests de résistances génotypiques qui avaient montré 45% de résistances aux INTI, 40% de résistances aux IP mais aussi des multi résistances simultanées à ces deux classes thérapeutiques dans 30% des cas. Les résistances aux INTI avaient montré les mutations suivantes dominées par la mutation M184V (63%) suivie de la mutation K65R (61%) puis de la mutation Q151M (11%), les associations K65R +M184V avaient concerné 10% des cas, la Q151M+ M184V (16%) et la Q151M+K65R+M184V (5%). Quant aux résistances aux IP les principales mutations retrouvées étaient I54M (27%), L90M (7%) et V47A (5%), des associations I54M+I82F (12%), I54M+L90M (6%) et I82F+L90M (6%). Au terme du suivi, la létalité globale a été de 23%: 3% à M12, 8% entre M12 et M24, 2% entre M24 et M48, 3% entre M48 et M60. Le taux de perdus de vus (PDV) était estimé à 34%: 10% à M12, 3% de M12 à M24, 3% entre M24 et M48 et 4% entre M48 et M60. Chez les patients non prétraités, la létalité a été de 16%: 3% à M12, 2% entre M12 à M24, 4% entre M24 et M48 et de 3% entre M48 et M60. Quant au taux de PDV, il était estimé à 30%: 9% à M12 et 6% entre M12 et M24, 4% entre M24 et M48 et 3% entre M48 et M60.

## Discussion

Notre étude a concerné 110 patients infectés par le VIH-2 sous traitement antirétroviral dont 68 patients non prétraités recevant le traitement de 1^ère^ ligne pour la première fois, suivis au service des maladies infectieuses de Fann, Dakar, Sénégal. Une prédominance féminine a été retrouvée dans notre série avec un sex ratio de 2,54. Ce constat corrobore la féminisation de l'épidémie du fait de la vulnérabilité de la femme en Afrique subsaharienne mais aussi pourrait s'expliquer par l'accès au dépistage plus étendu chez les femmes avec la recommandation systématique du dépistage au VIH au cours du suivi de la grossesse [13]. Cette prédominance féminine a été décrite dans l'étude de Ndour [14] au Sénégal et de Belestre [15] en Côte d'Ivoire mais à des proportions moins importantes. L'âge médian de notre série est de 46 ans. Cet âge médian est légèrement inférieur à celui qui a été retrouvé dans la sous-région [16]. Cette médiane d'âge est nettement supérieure à celle retrouvée dans l'infection à VIH-1 qui touche l'adulte plus jeune [14, 17]. Ce fait pourrait s'expliquer par la survie plus longue de l'infection à VIH-2 par rapport au VIH-1 et par sa longue phase asymptomatique [3, 4].

Sur le plan clinique, la plupart de nos patients consultaient au stade tardif avec une immunodépression sévère associée à plusieurs infections opportunistes. Ce recours tardif au soins est retrouvé dans plusieurs études africaines [12, 17, 18] mais aussi dans celles du nord [19-22]. Cette prévalence élevée du recours tardif suggère la nécessite de renforcer les pratiques de dépistage surtout dans le contexte de pays à épidémie concentrée où le dépistage est ciblé vers les groupes les plus exposés. Sur le plan thérapeutique, la classique association 2INTI à 1IP avait été règle dans notre étude dans 96% des cas, ce choix est conforme aux recommandations de l'OMS [10] et repose essentiellement sur le coût mais aussi sur la nécessité de maintenir des stocks de LPV/R générique pour le traitement de deuxième ligne de l'infection à VIH-1 [23]. Aux débuts de l'ISAARV, seul l'IP non boosté: l'Indinavir (IND) était disponible au Sénégal, à son retrait de l'ISAARV en 2008, des études ont montré qu'il est inactif contre le VIH- 2 du fait de sa concentration inhibitrice médiane IC50 élevée [24]. Sur le plan immunologique, notre étude avait montré une efficacité immunologique avec un gain de lymphocytes CD4 annuel de plus de 100 cellules/mm^3^. Cette efficacité immunologique a été retrouvée dans plusieurs études parmi lesquelles celles de Van Der Ende, Peterson et Bénard [25-27]. Par contre, d'autres études ont montré une efficacité immunologique moindre avec un gain inférieur à 100 cellules/ mm3 après un an de traitement antirétroviral [19, 28]. Une efficacité immunologique en faveur des IP boostés a été aussi décrite dans l'étude de Belestre [15].

Sur le plan virologique, contrairement à l'infection à VIH-1 où l'utilisation de la charge virale pour évaluer la réponse antirétrovirale est une recommandation de l'OMS adoptée dans plusieurs pays à ressources limitées [10], pour l'infection par le VIH-2, le plus souvent, ce sont des techniques internes de quantification de la charge virale qui sont actuellement utilisées avec des seuils allant de 10 à 1000 copies/ml [9, 11, 29-31]. Dans notre série, ce paramètre virologique a pu être évalué grâce à la collaboration américaine avec une technique maison utilisée par l'université de Washington, technique qui fixe un seuil d'échec virologique à partir de 50 copies/ml [11]. De ce fait, il existe en Afrique peu de données concernant les échecs virologiques et la prévalence retrouvée dans notre série est supérieure à celle qui a été décrite par Peterson et Diallo en Gambie [26, 32] et par Ekouevi en Côte d'Ivoire [16]. Elle est superposable à celle retrouvée par Smith [33], Raugi [12] et Gottlieb [34]. L'évaluation de la réponse virologique en fonction du schéma thérapeutique dans notre série avait montré une réponse moindre chez les patients qui étaient sous régime à base d'inhibiteurs de la protéase non boostés ou à base de trois inhibiteurs nucleéosidiques de la transcriptase inverse. Ce constat corrobore ce qui a été décrit par Bénard et Ruelle [20, 21]. Ces échecs virologiques avaient été associés à des résistances aux INTI et aux IP. Concernant les résistances aux INTI, les mutations les plus rencontrées étaient les mutations M184V, Q151M et K65R. Ces mutations, incriminées dans les résistances aux 3TC, TDF, AZT, D4T DDI et ABC ont été aussi décrites en Côte d'ivoire [35] mais aussi en Espagne [36].

Quant aux mutations associées aux résistances génotypiques aux IP, elles avaient été dominées par les mutations I54M, L90M et V47A. Ces mutations surtout combinées ont été décrites dans plusieurs études comme conférant une résistance élevée aux SQV, LPV/R , DRV mais aussi à l'IND [12, 27, 32, 37-39]. Ce qui rend problématique la prise en charge de l'infection à VIH-2 dans un contexte de pays à ressources limitées. En effet, aux débuts de l'ISARV, l'Indinavir a été le seul IP disponible jusqu'à son retrait en 2008, date ou son inactivité a été documentée (24). Cette inactivité de l'Indinavir explique la réponse thérapeutique moindre des patients qui étaient à ce régime par rapport au régime à base d'IP boosté comme le LPV/R. À l'heure actuelle, le LPV/R reste elle aussi le seul IP recommandé pour le traitement de première ligne de l'infection à VIH 2 (10). Cependant le V47A decrit dans notre étude, même seul confère une résistance au LPV/R [39, 40]. Ces constats suggèrent donc la nécessité de mener des essais randomisées pour déterminer les molécules les mieux appropriées pour le traitement de première ligne de l'infection à VIH-2 [5, 6]. Dans le même ordre d'idée, il serait pertinent de mener une surveillance épidémiologique des résistances en réalisant fréquemment des tests de génotypage qui pourront aider dans le choix des molécules efficaces pour le traitement de l'infection à VIH-2. En outre, il s'avère urgent de rendre accessibles les nouvelles classes thérapeutiques à savoir des IP boostés puissants et les inhibiteurs d'integrase pour faire face au traitement de deuxième ligne du patient infecté par le VIH-2. Ce d'autant plus, les recommandations sénégalaises, en cas d'échec de traitement de première ligne sont à l'heure actuelle, d'utiliser l'Atazanavir boosté (ATV/R) [10]. Cependant, du fait de l'inefficacité de l'ATV/R contre le VIH-2 documentée dans plusieurs études [38, 41] mais aussi du fait de l'arsenal thérapeutique dirigé contre le VIH-2 limité, nos patients avaient été maintenus sous le régime initial en attendant la disponibilité des nouvelles classes thérapeutiques efficaces. Ainsi, un taux de mortalité et de perdus de vue élevé avait été retrouvé dans notre série. Cette faible rétention aux soins a été aussi décrite par Tchounga et Peterson [18, 26].

## Conclusion

Il est ressorti de notre étude que la réponse à la trithérapie antirétrovirale chez le patient infecté par le VIH-2 est emmaillée d'échecs virologiques et de résistances génotypiques aux INTI et aux IP. De nos jours, il est donc impératif de rendre disponibles dans les pays à ressources limitées, les nouvelles classes thérapeutiques à savoir les autres IP boostés et les inhibiteurs d'integrase pour assurer le traitement de sauvetage des patients en échec thérapeutique. En plus, il s'avère nécessaire de réaliser des essais contrôlés randomisés pour mieux documenter les schémas thérapeutiques antirétroviraux optimaux pour le traitement de l'infection à VIH-2

### État des connaissances actuelles sur le sujet

- Le VIH-2 est endémique en Afrique de l'Ouest, il est naturellement résistant aux INNTI, l'OMS recommande d'utiliser dans les pays à ressources limitées 3 INTI et 1 IP comme traitement de première intention.

### Contribution de notre étude à la connaissance

- Notre étude a montré que la réponse de la trithérapie antirétrovirale classique contre le VIH-2 (2INTI et 1 IP) est entachée d'échec virologique et de résistances génotypiques, il est nécessaire aujourd'hui de rendre accessibles au Sénégal les inhibiteurs d'integrase et les nouveaux IP boostés pour le traitement de seconde ligne.

## Conflits d’intérêts

Les auteurs ne déclarent aucun conflit d’intérêts.
